# Feasibility and safety of robotic-assisted total pancreatectomy: a pilot western series

**DOI:** 10.1007/s13304-021-01079-3

**Published:** 2021-05-19

**Authors:** Emanuele F. Kauffmann, Niccolò Napoli, Valerio Genovese, Michael Ginesini, Cesare Gianfaldoni, Fabio Vistoli, Gabriella Amorese, Ugo Boggi

**Affiliations:** 1 Division of General and Transplant Surgery, University of Pisa, Pisa, Italy; 2 Division of Anesthesia and Intensive Care, Azienda Ospedaliera Universitaria Pisana, Pisa, Italy

**Keywords:** Total pancreatectomy, Robotic total pancreatectomy, Robotic pancreatectomy, Robotic pancreatoduodenectomy, Surgical complications, Pancreatic cancer

## Abstract

This study was designed to demonstrate non-inferiority of robot-assisted total pancreatectomy (RATP) to open total pancreatectomy (OPT) based on an intention-to-treat analysis, having occurrence of severe post-operative complications (SPC) as primary study endpoint. The two groups were matched (2:1) by propensity scores. Assuming a rate of SPC of 22.5% (non-inferiority margin: 15%; *α*: 0.05; *β*: 0.20; power: 80%), a total of 25 patients were required per group. During the study period (October 2008–December 2019), 209 patients received a total pancreatectomy. After application of exclusion and inclusion criteria, matched groups were extracted from an overall cohort of 132 patients (OPT: 107; RATP: 25). Before matching, the two groups were different with respect to prevalence of cardiac disease (24.3% versus 4.0%; *p* = 0.03), presence of jaundice (45.8% versus 12.0%; *p* = 0.002), presence of a biliary drainage (23.4% versus 0; *p*  = 0.004), history of weight loss (28.0% versus 8.0%; *p* = 0.04), and vein involvement (55.1% versus 28.0%) (*p* = 0.03). After matching, the two groups (OTP: 50; RATP: 25) were well balanced. Regarding primary study endpoint, SPC developed in 13 patients (26.0%) after OTP and in 6 patients (24.0%) after RATP (*p* = 0.85). Regarding secondary study endpoints, RATP was associated with longer median operating times [475 (408.8–582.5) versus 585 min (525–637.5) *p* = 0.003]. After a median follow-up time of 23.7 months (10.4–71), overall survival time [22.6 (11.2–81.2) versus NA (27.3–NA) *p* = 0.006] and cancer-specific survival [22.6 (11.2–NA) versus NA (27.3–NA) *p* = 0.02] were improved in patients undergoing RATP. In carefully selected patients, robot-assisted total pancreatectomy is non-inferior to open total pancreatectomy regarding occurrence of severe post-operative complications.

**Supplementary Information:**

The online version contains supplementary material available at 10.1007/s13304-021-01079-3.

## Introduction

Total pancreatectomy was initially introduced to avoid the consequences of post-operative pancreatic fistula [1] and to improve surgical radicality [2]. The procedure became very popular in the late ‘70s and in the ‘80s, when over 40% of pancreatic resections were total pancreatectomies [3]. Enthusiasm, however, was soon mitigated by the evidence that total pancreatectomy neither reduced post-operative mortality [4] nor improved long-term survival [5]. Additionally, total pancreatectomy decreases quality of life, by creating full exocrine and endocrine insufficiency [6]. Currently, total pancreatectomy is performed selectively, in approximately 6% of the patients who receive a pancreatic resection [7]. In the majority of these patients, total pancreatectomy is required due to widespread involvement of the gland [8] or in case of locally advanced tumors requiring arterial resection and reconstruction [9]. These indications may not apply well to a minimally invasive approach. However, total pancreatectomy may also be required in patients with positive frozen section histology of pancreatic neck margin during pancreaticoduodenectomy [10], multifocal endocrine tumors [11, 12], metastatic tumors [13], main duct intraductal papillary mucinous tumors [12, 14], premalignant lesions in patients with history of familial pancreatic cancer [8], extremely soft pancreatic remnants and small ducts in patients with right-sided tumors [15], and chronic pancreatitis with refractory pain [16]. In these patients, a minimally invasive approach may be an appealing alternative to open surgery. However, laparoscopic total pancreatectomy continues to be performed rarely. Barriers to wider adoption of laparoscopy for total pancreatectomy are likely to include the need for extensive retroperitoneal dissection while handling a rather bulky specimen in a deep space, the need to construct two digestive anastomoses, and the necessity to introduce several adaptations in respect to well-established open surgical techniques. At least in theory, the use of robotic assistance could facilitate the performance of all these tasks and could result in faster and safer implementation of minimally invasive total pancreatectomy.

In 2015, we reported on 11 cases of robotic-assisted total pancreatectomy (RATP) in the context of a matched analysis with open total pancreatectomy (OTP). In this study, we showed that RATP was feasible, despite longer operating times, and was associated with reduced blood loss, earlier achievement of independent mobility, earlier recovery of bowel functions, and improved pain scores with proportionally reduced need for post-operative analgesic therapy [17].

We herein present on 25 RATPs and provide a further comparison with contemporary cohort of OTPs matched by propensity scores.

## Methods

This study was designed and reported according to the STROBE guidelines [18], and was approved by the Institutional Review Board of our hospital. Data were extracted from a prospectively maintained database and were analyzed retrospectively.

This study includes patients operated between October 2008 and December 2019 at the Division of General and Transplant Surgery of the Azienda Ospedaliera Universitaria Pisana, serving as the main referral center for surgical treatment of pancreatic diseases for approximately 1.2 million people, and receiving also patient referrals from all over Italy. At our Institution, the first da Vinci Surgical System (Intuitive Surgical^®^, Sunnyvale, California, US) was installed in the year 2000.

Selection criteria for RATP were: general suitability for laparoscopic surgery, absence of history of previous major upper abdominal surgery (e.g., partial or total gastrectomy, or major hepatectomy), body mass index ≤ 35 kg/m^2^, a preoperative diagnosis requiring total pancreatectomy while excluding locally advanced malignancy, and timely availability of the robot. Vein involvement without severe stenosis or obstruction was not considered an absolute contraindication.

To confirm that RATP is feasible and to further investigate its possible advantages, a control group was selected from a contemporary cohort of patients undergoing total pancreatectomy by an open approach.

To minimize bias from nonrandomized treatment assignment, RATP cases were matched with a 2:1 ratio with OTP controls using a conservative caliper width of 0.2. The following parameters, known to predict post-operative outcomes in total pancreatectomy, were used: age [19, 20], gender [19, 20], body mass index [21], history of cardiac disease [22], American Society of Anesthesiologists (ASA) score [23, 24], history of weight loss [25], presence of jaundice [26], preoperative biliary drainage [27, 28], and involvement of the superior mesenteric/portal vein [23, 28].

The technique for RATP was previously described in detailed [17], and is shown in the attached video (supplementary material 1), but some important tips and tricks deserve a specific comment. First, in case of concurrent splenectomy, the left gastric vein should be preserved to avoid gastric congestion [29]. Second, when the spleen can be spared, preservation of the splenic vessels (alike in a Kimura procedure) is preferable [30] to avoid sinistral portal hypertension, but the lymph nodes located along the splenic vessels should be removed to ensure adequate staging [31]. Third, the first duodenal portion (or gastric antrum) should not be divided until the distal pancreas are fully mobilized to prevent retraction of the stomach in the left upper abdominal quadrant [17]. Fourth, during retroperitoneal dissection, large lymphatics should be sealed by ligatures or clips [32] to reduce incidence and severity of prolonged lymphatic drainage, including chyle leak [33].

In patients requiring vein resection and reconstruction, the vascular procedure was classified according to the International Study Group of Pancreatic Surgery [34].

### Definition of study outcomes

Post-operative complications were graded using the Clavien–Dindo classification [35], and were considered severe when ≥ 3. The comprehensive complication index (CCI) was also calculated [36]. Delayed gastric emptying (DGE) [37] and postpancreatectomy hemorrhage (PPH) [38] were defined according to the International Study Group on Pancreatic Surgery. Post-operative death, was defined as any death occurring during the initial hospital stay or the first 90 days after surgery.

Overall survival (OS), disease-free survival (DFS), and cancer-specific survival (CCS) were defined according to DATECAN (Definition for Assessment of Time-to-event End-points in CANcer trials) [39].

### Primary and secondary study endpoints

The primary endpoint of this study was the rate of severe post-operative complications.

Secondary endpoints were: operating time, length of hospital stay, proportion of patients with grade 1–2 post-operative complications, CCI score, proportion of patients receiving blood transfusions, incidence and severity of PPH, incidence and severity of DGE, need for repeat surgery at 90 days, number of examined lymph nodes, proportion of patients receiving adjuvant chemotherapy, proportion of patients completing adjuvant chemotherapy, OS, DFS, and CSS.

### Follow-up

After hospital discharge patients were seen in our outpatient clinic at least once a month during the first 3 months and every 3 months thereafter. A computed tomography scan was obtained every 6 months for the first 5 years, and yearly thereafter.

### Statistical analysis

The study was designed to demonstrate non-inferiority of RATP to OPT in terms of occurrence severe post-operative complications based on an intention-to-treat analysis that keeps in the minimally invasive group the patients who required conversion to open surgery. An overall rate of 22.5% [22] was assumed for both groups, and the non-inferiority margin was set at 15%. In addition, the *α* was set at 0.05 and *β* at 0.20, yielding a power of 80%. Therefore, to demonstrate non-inferiority of experimental (i.e., RATP) and standard (i.e., OTP) a total of 50 patients would be required to be 80% certain that the upper limit of a one-sided 97.5%CI—or equivalently a two-sided 95%CI—would exclude a difference in favor of OPT exceeding 15%.

Quantitative variables are presented as mean ± SD if normally distributed, or as median and interquartile range (IQR) if not. Categorical variables are expressed as frequencies, percentages, and rates. Kolmogorov–Smirnov test was used to assess normality distribution. Chi-square test was used to evaluate the presence of an association between surgical technique (OPD and RPD) and outcome.

All statistical analyses were carried out with JMP^®^ 15.2.0 software package for Mac, Copyright© SAS Institute Inc., SAS campus Drive, Cary, NC, USA and R Package, R Core Team (2014): A language and Environment for Statistical Computing, R Foundation for Statistical Computing, Vienna AT using *Matching*, *MatchIt* and *TrialSize* as packages.

## Results

**Fig. 1:**
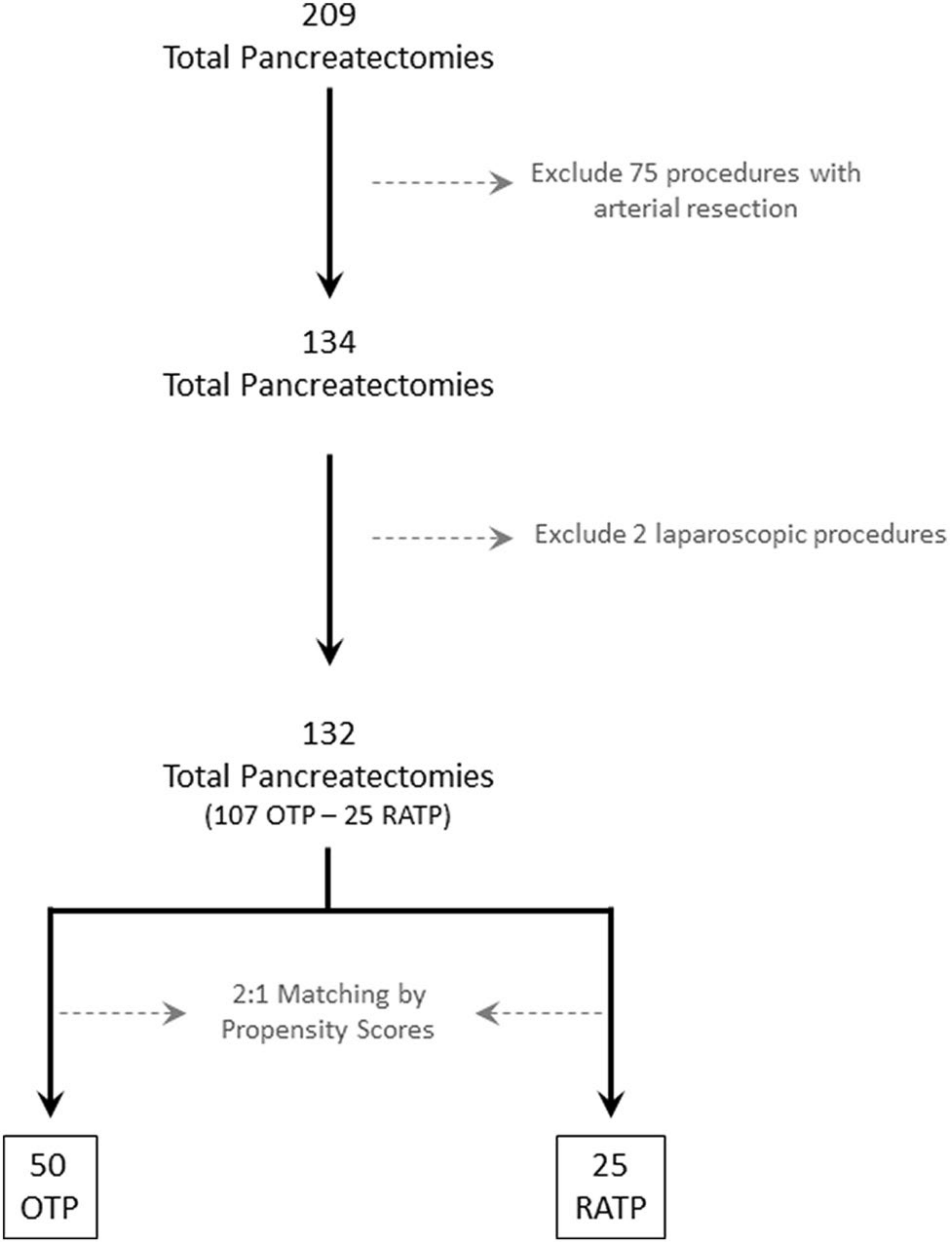
Study flowchart

During the study period, a total of 209 patients underwent total pancreatectomy. After exclusion of 75 patients with concurrent arterial resection and 2 patients operated laparoscopically, there were 132 patients who received either an OTP (*n* = 107) or a RATP (*n* = 25). After matching, there were 50 OTP and 25 RATP (Fig. 1).

Before matching, the two groups differed in prevalence of cardiac disease (1; 4.0% versus 26; 24.3%) (*p* = 0.03), presence of jaundice (3; 12.0% versus 49; 45.8%) (*p* = 0.002), need for a biliary drainage (0 versus 25; 23.4%) (*p* = 0.004), history of weight loss (2; 8.0% versus 30; 28.0%) (*p* = 0.04), and vein involvement (7; 28.0% versus 59; 55.1%) (*p* = 0.03).

The matching process identified 75 patients, including 50 OTPs and 25 RATPs. As shown in Table 1, the two groups were fully matched for all baseline characteristics.

**Table 1:** Baseline characteristics before and after 2:1 matching by propensity scores

	Before matching				After matching			
	All	RATP	OTP	*p*	All	RATP	OTP	*p*
Number of patients	132 (100%)	25 (18.9%)	107 (81.1%)	NA	75 (100%)	25 (33%)	50 (66.7%)	NA
Age; years; mean ± SD	67.2 ± 10	66.7 ± 8.9	67.1 ± 1	0.79	67.5 ± 9.5	67.7 ± 8.9	67.4 ± 9.9	0.9
Female gender; number (%)	52 (39.4%)	12 (48%)	40 (37.4%)	0.33	33 (44%)	12 (48%)	21 (42%)	0.62
BMI; kg/m^2^; mean ± SD	24.6 ± 3.3	23.7 ± 2.2	24.8 ± 0.3	0.17	23.8 ± 2.5	23.7 ± 2.2	23.8 ± 2.6	0.85
ASA score; median (IQR)	3 (2–3)	3 (2–3)	3 (3–3)	0.15	3 (2–3)	3 (2–3)	3 (2–3)	0.71
Cardiac disease; number (%)	27 (20.5%)	1 (4%)	26 (24.3%)	**0.03**	6 (8%)	1 (4%)	5 (10%)	0.66
COPD; number (%)	15 (11.4%)	3 (12%)	12 (11.2%)	1	7 (9.3%)	3 (12%)	4 (8%)	0.68
Hypertension; number (%)	65 (49.2%)	14 (56%)	51 (47.7%)	0.45	35 (46.7%)	14 (56%)	21 (42%)	0.25
Diabetes mellitus; number (%)	49 (37.1%)	10 (40%)	39 (36.5%)	0.74	26 (34.7%)	10 (40%)	16 (32%)	0.49
Jaundice; number (%)	52 (39.4%)	3 (12%)	49 (45.8%)	**0.002**	12 (16%)	3 (12%)	9 (18%)	0.74
Abdominal pain; number (%)	66 (50%)	12 (48%)	54 (50.5%)	0.82	35 (46.7%)	12 (48%)	23 (46%)	0.87
Duodenal obstruction; number (%)	9 (6.8%)	2 (8%)	7 (6.5%)	0.68	5 (6.7%)	2 (8%)	3 (6%)	1
Weight loss; number (%)	32 (24.2%)	2 (8%)	30 (28%)	**0.04**	100 (13.3%)	2 (8%)	8 (16%)	0.48
Vein involvement; number (%)	66 (50%)	7 (28%)	59 (55.1%)	**0.03**	31 (41.3%)	7 (28%)	24 (48%)	0.10
Biliary drainage, number (%)	25 (18.9%)	0 (0%)	25 (23.4%)	**0.004**	0 (0%)	0 (0%)	0 (0%)	0
Previous abdominal surgery; number (%)	79 (59.8%)	15 (60%)	64 (59.8%)	0.99	46 (61.3%)	15 (60%)	31 (62%)	1
Preoperative chemotherapy; number (%)	9 (6.8%)	0 (0%)	9 (8.4%)	0.21	3 (4%)	0 (0%)	3 (6%)	0.55

Statistically significant *p* values are highlighted in bold

*BMI* body mass index, *ASA* American Society of Anesthesiologists, *COPD* chronic obstructive pulmonary disease

Fifteen RATPs (60%) were two-stage procedures (i.e., resection was extended to achieve a radical resection following positive frozen section histology at the neck margin). The proportion of two-stage procedures was similar in the two groups (60% vs 48.6%; *p* = 0.30).

Conversion to open surgery was required in two RATPs (8.0%). In both patients, conversion occurred under elective conditions because of unanticipated need for multivisceral resection and chronic inflammation of peripancreatic tissues precluding safe dissection, respectively.

As shown in Table 2, RATPs was associated with longer operating time, higher rates of pylorus preservation, and lower rates of type 3 vein reconstruction in both unmatched and matched cohorts. Before matching differences were also noted regarding the need for vein resection. Looking specifically at operative parameters in matched cohorts, it is worth to note that the main difference between the two groups was longer median operating time in RATPs (585; 525–637.5 min versus 475; 408.8–582.5 min) (*p* = 0.0003). Differences in rates of pylorus preservation (23; 92.0% versus 35; 70.0%) (*p* = 0.04), and rate of type 3 venous reconstruction (3; 12.0% versus 18; 36.0%) (*p* = 0.03) were also noted.

**Table 2:** Operative time and procedures

	Before matching				After matching			
	All	RATP	OTP	*p*	All	RATP	OTP	*p*
Number of patients	132 (100%)	25 (18.9%)	107 (81.1%)	NA	75 (100%)	25 (33.%)	50 (66.7%)	NA
Operative time; minutes; median (IQR)	520 (445–610)	585 (525–637.5)	500 (430–600)	**0.002**	520 (445–605)	585 (525–637.5)	475 (408.8–582.5)	**0.0003**
En-bloc splenectomy (%)	107 (81.1%)	18 (72%)	89 (83.2%)	0.20	60 (80%)	18 (72%)	42 (84%)	0.22
Preservation of spleen and splenic vessels; number (%)	25 (18.9%)	7 (28%)	18 (16.8%)	0.20	15 (20%)	7 (28%)	8 (16%)	0.22
Pylorus preservation; number (%)	91 (68.9%)	23 (92%)	68 (63.6%)	**0.007**	58 (77.3%)	23 (92%)	35 (70%)	**0.04**
Total gastrectomy; number (%)	6 (4.5%)	0 (0%)	6 (5.6%)	0.59	2 (2.7%)	0 (0%)	2 (4%)	0.55
Multivisceral resection; number (%)	27 (20.5%)	2 (8%)	25 (23.4%)	0.1	16 (21.3%)	2 (8%)	14 (28%)	0.07
Vein resection; number (%)	66 (50%)	7 (28%)	59 (55.1%)	**0.03**	31 (41.3%)	7 (28%)	24 (48%)	0.10
Superior mesenteric vein resection; number (%)	11 (8.3%)	0 (0%)	11 (10.3%)	0.12	5 (6.7%)	0 (0%)	5 (10%)	0.16
Portal vein resection; number (%)	3 (2.3%)	2 (8%)	1 (0.9%)	0.09	2 (2.7%)	2 (8%)	0 (0%)	0.11
Mesenteric-portal confluence resection; number (%)	52 (39.4%)	5 (20%)	47 (43.9%)	**0.03**	24 (32%)	5 (20%)	19 (38%)	0.12
Type 3 vein reconstruction; number (%)	48 (36.4%)	3 (12%)	45 (42.1%)	**0.005**	21 (28%)	3 (12%)	18 (36%)	**0.03**
Type 4 vein reconstruction; number (%)	18 (13.6%)	4 (16%)	14 (13.1%)	0.74	10 (13.3%)	4 (16%)	6 (12%)	0.72

Statistically significant *p* values are highlighted in bold

Regarding the main endpoint of this study, as shown in Table 3, severe post-operative complications developed in 6 patients (24.0%) after RATP and in 13 patients (26.0%) after OTP (*p* = 0.85). Overall, there were 9 post-operative deaths (6.8%) in unmatched cohorts, and 5 (6.7%) in matched cohorts with 1 death after RATP (4.0%) and 4 deaths after OTP (8.0%) (*p* = 0.66).

**Table 3:** Post-operative results

	Before matching				After matching			
	All	RATP	OTP	*p*	All	RATP	OTP	*p*
Number of patients	132 (100%)	25 (18.9%)	107 (81.1%)	NA	75 (100%)	25 (33%)	50 (66.7%)	NA
Length of stay; median (IQR); days	20 (15–30)	22 (14.5–30.5)	19 (15–30)	0.54	20 (14–28)	22 (14.5–30.5)	18 (14–28)	0.19
Patients receiving blood transfusions; number (%)	40 (30.3%)	5 (20%)	35 (32.7%)	0.21	24 (32%)	5 (20%)	19 (38%)	0.12
Patients without complications; number (%)	42 (31.8%)	7 (28%)	35 (32.7%)	0.65	27 (36%)	7 (28%)	20 (40%)	0.31
Patients with grade I complications; number (%)	3 (2.3%)	0 (0%)	3 (2.8%)	1	1 (1.3%)	0 (0%)	1 (2%)	1
Patients with grade II complications; number (%)	50 (37.9%)	12 (48%)	38 (35.5%)	0.25	28 (37.3%)	12 (48%)	16 (32%)	0.18
Patients with grade IIIa complications; number (%)	13 (9.8%)	4 (16%)	9 (8.4%)	0.27	7 (9.3%)	4 (16%)	3 (6%)	0.21
Patients with grade IIIb complications; number (%)	6 (4.5%)	0 (0%)	6 (5.6%)	0.59	4 (5.3%)	0 (0%)	4 (8%)	0.29
Patients with grade IVa complications; number (%)	8 (6.1%)	1 (4%)	7 (6.5%)	1	3 (4%)	1 (4%)	2 (4%)	1
Patients with grade IVb complications; number (%)	1 (0.8%)	0 (0%)	1 (0.9%)	1	0 (0.0%)	0 (0%)	0 (0%)	NA
Patients with grade V complications; number (%)	9 (6.8%)	1 (4%)	8 (7.5%)	1	5 (6.7%)	1 (4%)	4 (8%)	0.66
Patients with severe complications (≥ 3a); number (%)	37 (28%)	6 (24%)	31 (29%)	0.62	19 (25.3%)	6 (24%)	13 (26%)	0.85
Comprehensive Complication Index; median (IQR)	22.6 (0–33.7)	20.9 (0–32.5)	22.6 (0–36.2)	0.76	20.9 (0–29.6)	20.9 (0–32.5)	20.9 (0–39.6)	0.72
Post-pancreatectomy hemorrhage; number (%)	10 (7.6%)	2 (8%)	8 (7.5%)	1	7 (9.3%)	2 (8%)	5 (10%)	1
Grade A; number (%)	1 (0.8%)	0 (0%)	1 (0.9%)	1	0 (0.0%)	0 (0%)	0 (0%)	NA
Grade B; number (%)	4 (3%)	1 (4%)	3 (2.8%)	0.57	3 (4%)	1 (4%)	2 (4%)	1
Grade C; number (%)	5 (3.8%)	1 (4%)	4 (3.7%)	1	4 (5.3%)	1 (4%)	3 (6%)	1
Delayed gastric emptying; number (%)	14 (10.6%)	6 (24%)	8 (7.5%)	**0.02**	11 (14.7%)	6 (24%)	5 (10%)	0.11
Grade A; number (%)	0 (0%)	0 (0%)	0 (0%)	NA	0 (0%)	0 (0%)	0 (0%)	NA
Grade B; number (%)	11 (8.3%)	4 (16%)	7 (6.5%)	0.22	8 (10.7%)	4 (16%)	4 (8%)	0.43
Grade C; number (%)	3 (2.3%)	2 (8%)	1 (0.9%)	0.09	3 (4%)	2 (8%)	1 (2%)	0.26
Biliary leak; number (%)	3 (2.3%)	0 (0%)	3 (2.8%)	1	1 (1.3%)	0 (0%)	1 (2%)	1
Enteric fistula; number (%)	5 (3.8%)	0 (0%)	5 (4.5%)	0.58	3 (4%)	0 (0%)	3 (6%)	0.55
Medical complications; number (%)	71 (53.8%)	11 (44%)	60 (56.1%)	0.28	35 (46.7%)	11 (44%)	24 (48%)	0.74
Repeat surgery at 90 days; number (%)	17 (12.9%)	2 (8%)	15 (14%)	0.53	9 (12%)	2 (8%)	7 (14%)	0.71

Statistically significant *p* value is highlighted in bold

**Fig. 2:**
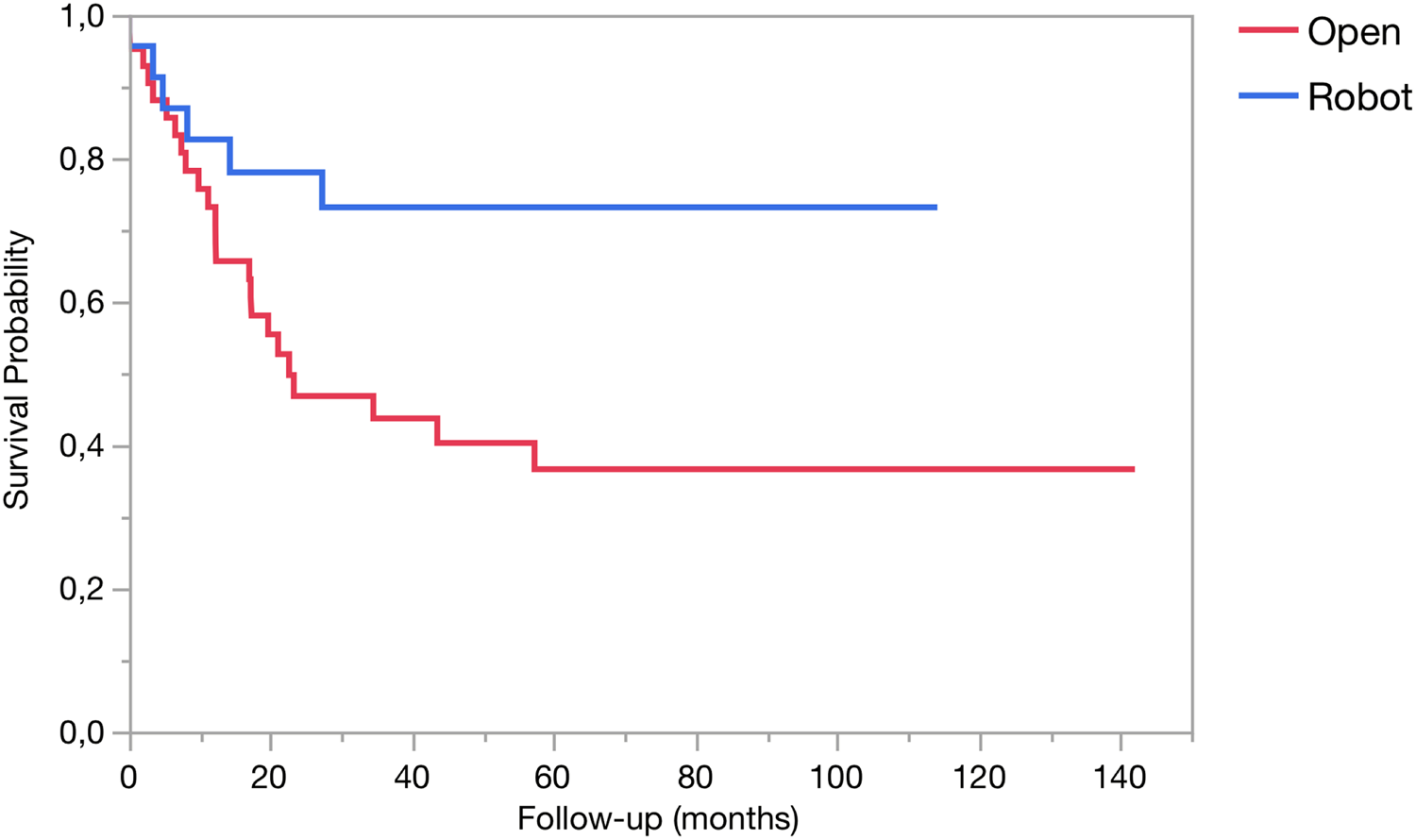
Kaplan–Meier curves for cancer-specific survival for matched patients undergoing either OPD (red line) or RATP (blue line) for pancreatic cancer or malignant IPMN

Regarding the secondary endpoints of this study, differences in operating times were already presented. Concerning the remaining parameters, results achieved in the two study groups were equivalent with respect to length of hospital stay, proportion of patients with grade 1–2 post-operative complications, CCI score, proportion of patients receiving blood transfusions, incidence and severity of PPH, incidence and severity of DGE, need for repeat surgery at 90 days, number of examined lymph nodes, proportion of patients receiving adjuvant chemotherapy, proportion of patients completing adjuvant chemotherapy, and DFS (Tables 3, 4, and 5). RATPs was instead associated with longer overall OS and CSS (Fig. 2).

**Table 4:** Pathology of resected specimens

	Before matching				After matching			
	All	RATP	OTP	*p*	All	RATP	OTP	*p*
Number of patients	132 (100%)	25 (18.9%)	107 (81.1%)	NA	75 (100%)	25 (33%)	50 (66.7%)	NA
**Tumor types**								
Pancreatic ductal adenocarcinoma; number (%)	45 (34.1%)	5 (20%)	40 (37.4%)	0.10	20 (26.7%)	5 (20%)	15 (30%)	0.42
Malignant IPMN; number (%)	47 (35.6%)	11 (44%)	36 (33.6%)	0.33	26 (34.7%)	11 (44%)	15 (30%)	0.23
IPMN; number (%)	19 (14.4%)	7 (28%)	12 (11.2%)	0.03	17 (22.7%)	7 (28%)	10 (20%)	0.44
Ampullary carcinoma; number (%)	2 (1.5%)	0 (0%)	2 (1.9%)	1	0 (0%)	0 (0%)	0 (0%)	NA
Duodenal adenocarcinoma; number (%)	1 (0.8%)	0 (0%)	1 (0.9%)	1	1 (1.3%)	0 (0%)	1 (2%)	1
Acinar cell carcinoma; number (%)	1 (0.8%)	0 (0%)	1 (0.9%)	1	0 (0%)	0 (0%)	0 (0%)	NA
Gastric cancer; number (%)	1 (0.8%)	0 (0%)	1 (0.9%)	1	0 (0%)	0 (0%)	0 (0%)	NA
Metastasis from renal cell carcinoma; number (%)	6 (4.5%)	1 (4%)	5 (4.7%)	1	5 (6.7%)	1 (4%)	4 (8%)	0.66
Chronic pancreatitis; number (%)	6 (4.5%)	1 (4%)	5 (4.7%)	1	3 (4%)	1 (4%)	2 (4%)	1
Neuroendocrine tumor; number (%)	3 (2.3%)	0 (0%)	3 (2.8%)	1	2 (2.7%)	0 (0%)	2 (4%)	1
Lymphoma; number (%)	1 (0.8%)	0 (0%)	1 (0.9%)	1	1 (1.3%)	0 (0%)	1 (2%)	1
Number of examined lymph nodes; mean ± SD	63.3 ± 27.2	66.1 ± 5.5	62.6 ± 2.6	0.57	61 ± 25.8	66.1 ± 5.5	58.4 ± 24	0.26
Number of positive lymph nodes; median (IQR)	1 (0–4)	0 (0–1)	1 (0–5)	0.02	0 (0–3)	0 (0–1)	0 (0–5.3)	0.25
Patients with positive margins^a^; number (%)	30 (30.9%)	4 (25%)	26 (32.1%)	0.77	14 (29.8%)	4 (25%)	10 (32.3%)	0.74
Patients with confirmed vascular invasion; number (%)	42 (63.6%)	4 (57.1%)	38 (64.4%)	0.70	22 (71%)	4 (57.1%)	18 (75%)	0.38
Length of resected vein segment; cm; mean ± SD	3.1 ± 1.1	2.1 ± 0.5	3.2 ± 1.1	**0.009**	2.8 ± 1	2.1 ± 0.5	2.9 ± 1	**0.04**

Statistically significant *p* values are highlighted in bold

*IPMN* intraductal papillary mucinous neoplasm

^a^Margins are assessed circumferentially and at 1 mm

**Table 5:** Oncologic follow-up in patients with pancreatic cancer and malignant IPMN (not including post-operative deaths)

	Before matching				After matching			
	All	RATP	OTP	*p*	All	RATP	OTP	*p*
Patients number (%)	83 (62.9%)	15 (60%)	68 (63.6%)	0.74	41 (54.7%)	15 (60%)	26 (52%)	0.51
Patients receiving adjuvant chemotherapy; number (%)	57 (60.6%)	7 (46.7%)	50 (63.3%)	0.23	26 (56.5%)	7 (46.7%)	19 (61%)	0.35
Patients completing adjuvant chemotherapy; number (%)	43 (62.3%)	9 (75%)	34 (59.7%)	0.32	22 (66.7%)	9 (75%)	13 (61.9%)	0.44
Overall survival; months; median (IQR)	27.3 (10.2–NA)	NA (27.3–NA)	23.3 (9.8–NA)	**0.004**	43.5 (12.2–NA)	NA (27.3-NA)	22.6 (11.2–81.2)	**0.006**
Cancer specific survival; months; median (IQR)	27.3 (10.2–NA)	NA (27.3–NA)	23.3 (9.8–NA)	**0.006**	NA (12.2–NA)	NA (27.3-NA)	22.6 (11.2-NA)	**0.02**
Disease-free survival; months; median (IQR)	9.1 (4.5–15.5)	7.6 (7.6–7.6)	10.1 (4–16)	0.63	10.5 (5.5–19.07)	7.6 (7.6–7.6)	11.2 (5.5–19.7)	0.58

Statistically significant *p* values are highlighted in bold

*RTPD* robot-assisted total pancreatoduodenectomy, *OTP* open total pancreaticoduodenectomy

Details on histology of resected specimens are reported and oncologic follow-up are reported in in Tables 4 and 5, respectively.

## Discussion

Total pancreatectomy is certainly a major procedure that, even when successful, imposes major consequences on patients resulting in impaired quality of life [6, 19]. Therefore, patients requiring a total pancreatectomy could not be considered good candidates for a minimally invasive procedure, which is typically performed to enhance patients’ rehabilitation [40, 41], improve quality of life [42, 43], and minimize the impact of surgery on body image [44, 45]. On the other hand, in properly selected patients, total pancreatectomy could be conveniently performed through a minimally invasive approach, because it does not require a pancreatic anastomosis and therefore avoids post-operative pancreatic fistula by definition. Additionally, a minimally invasive approach has the potential to reduce some complications that occur frequently after total pancreatectomy such as DGE [19], pulmonary complications [19], abdominal infections [46], and surgical site infections [23].

RATP is a surgical innovation, since it is a “*modified surgical procedure that differs from currently accepted local practice, the outcomes of which have not been described, and which may entail risk to the patient*” [47]. When classified according to the Idea, Development, Exploration, Assessment, Long Term Study (IDEAL) recommendations [48], RATP has nearly completed stage 2a (development) and is moving forward in stage 2b (exploration). Stage 2a refers to a procedure that may still require technical refinements and that has been performed only in small groups of patients. Stage 2b begins when the main technical aspects of the procedure have been fixed, but experience is still limited. In both stages, outcomes should be recorded prospectively to ensure that all adverse events are captured. Reporting in stage 2a includes selection criteria, proportion of eligible patients, technical modifications, clinical outcomes, and specific complications. Reporting in stage 2b should mostly consist of clinical studies providing preparatory information for subsequent major randomized clinical studies [48].

According to this background, this study provides the highest possible level of evidence for a procedure in stage 2a–2b in the IDEAL framework (i.e., RATP), by assessing safety (i.e., incidence of severe post-operative complications) in the context of a propensity score matched comparison with the current treatment standard (i.e., OTP). Our data show that RATP is non-inferior to OTP with respect to occurrence of SPC. The relevance of this piece of information is enhanced by the fact that our results were achieved in the first 25 RATPs. Despite the learning curve for robotic total pancreatectomy has not been defined yet, and could be influenced by contemporary volume of other types of pancreatic resections, it is reasonable to accept that with further experience, we should be able to reduce our operating times. As longer operating times were one of the main difference between RATP and OTP, and duration of surgery exceeding 420 min is a strong prognostic factor for the development of post-operative complications [19], future results could be more favorable.

### Technical considerations

Despite total pancreatectomy is almost automatically associated with the concept of splenectomy, we could preserve the spleen along with the splenic vessels in 18 of 107 OTPs (16.8%) and in 7 of 25 (28.0%) RATPs. Other series have shown rates of spleen preservation ranging from 6.4 to 34% [19, 20].

In general, spleen preservation is considered to be important to reduce intraoperative bleeding [49], to decrease the risk of thromboembolic events [50], and to prevent overwhelming post splenectomy spesis [51]. In patients diagnosed with pancreatic tumors, splenectomy does not improve oncologic radicality, mostly due to the rare occurrence of lymph-node metastasis at the splenic hilum [52, 53], and was instead shown to reduce long-term survival [54] as already shown for the stomach [55] and the colon [56]. Finally, several in-vitro studies have demonstrated that the spleen plays in antitumor immune response and that splenectomy could facilitate development of distant metastasis [57, 58]. The robotic approach is known to facilitate spleen preservation during distal pancreatectomy, especially using the Kimura technique [59]. Although the spleen can be preserved also when sacrificing the splenic vessels, alike in a Warshaw procedure [60], we do not favor this approach in total pancreatectomy, since spleen supply would be left to a collateral circulation based on the left gastric vessels alone.

Considering that pylorus preservation is standard at our Institution [61], the higher ability to meet this goal with RATP (92.0% versus 70.0%) might mean that the robotic assistance improves the ability to preserve blood supply to the entire stomach when extensive retroperitoneal dissection is required.

In general, in patients requiring a vein resection, we favor segmental over tangential vein resection [62, 63]. In our hands and in the open setting, most of these procedures are currently type 3 vein resections/reconstructions. In RATP, we noted fewer direct, end-to-end, vein reconstructions with a proportional increase in type 4 procedures. This can be readily explained by the need to place the patient in reverse Trendelenburg position [17] and the relative inability to perform a Cattell–Braasch maneuver [64]. Due to these challenges, other groups prefer to pursue type 1–2 vein resections/reconstructions during minimally invasive procedures [65–68]. While all technical solutions are acceptable, provided that the opportunity of achieving a radical resection is not missed and vein patency is maintained, we recommend that surgeons should have a clear strategy for vein resection and reconstruction before embarking upon these procedures. These strategies might be different from those established in the open setting.

### Length of hospital stay as a surrogate marker of early recovery

Most studies on minimally invasive procedures emphasize the importance of reduced length of hospital stay (versus open surgery). In some studies, especially from the United States, hospital stay may be very short, even in case of major procedures. In this study, we have not reported a short hospital stay after RATP. While achieving this goal is certainly important for stakeholders, it should be recognized that length of hospital stay may not be an objective parameter to evaluate the efficacy of surgical procedures, since it can be influenced by external factors, such as local and cultural attitudes [32]. Additionally, after total pancreatectomy, patients need to be trained to manage brittle diabetes before they can safely leave the hospital [69]. Finally, patients living far from hospital may feel unsecure if discharged too early, and may not accept this decision. We therefore suggest the use of more objective parameters, such as time to functional recovery [70], to show efficacy of post-operative recovery in surgical procedures.

### Other reports on RATP

An analysis of current literature demonstrates only few case reports [12, 71, 72] and small series [12, 14, 17, 46, 73] reporting on either laparoscopic total pancreatectomy or RATP. Excluding our previous study [17], there is only one additional study comparing matched cohorts of RATP and OTP. In this study, Weng and co-workers report on 15 RATP and 78 OTP performed over a period of slightly more than 4 years at a Chinese center performing an average over 1000 pancreatic resections per year [46]. In unmatched cohorts, the two groups differed in BMI (lower in RATP), incidence of vascular involvement (less frequent in RATP), presence of variations in arterial liver supply (less frequent in RATP), rate of spleen preservation (higher in RATP), median length of hospital stay (shorter in RATP), number of examined lymph nodes (lower in RATP), and operating time (shorter in RATP). After 1:1 matching by propensity scores all differences but shorter operating time in RATP disappeared. This study confirmed that RATP, in selected patients, is not associated with an increased risk of post-operative complications. Actually, these authors reported impressively low rates of SPC after either RATP (6.7%) or OTP (14.1%). These figures should be carefully interpreted as they refer to a follow-up of only 30 days and were achieved in the context of a patient population in which two-thirds of the patients were classified ≤ ASA 2. Additionally, it is not clear how this group could achieve shorter operating times in the robotic group, but their impressively high annual volumes of robotic pancreatic resections (approximately 300 procedures per year) reinforce the concept that with enough practice operating times can be significantly reduced. It is also worth to note that even if length of hospital stay was shorter in the robotic group (18 versus 20 days), these figures are similar to our results (22 versus 18 days). Also the rate of spleen preservation in the robotic group (26.7%) is similar to the one recorded in our series (28.0%), further reinforcing the concept that robotic assistance facilitates spleen preservation [46].

### Study limitations

This study has several limitations. First, despite prospective collection of data, the retrospective analysis carries the inherent risk of hidden biases mostly related to patient selection. Second, despite reporting on one of the largest series of minimally invasive total pancreatectomies, the relatively small number of procedures may not be sufficient enough to depict the full spectrum and severity of complications occurring following RATPs. Third, this series of RATPs was performed at a single institution, thereby limiting the generalizability of the results.

## Conclusions

In conclusion, despite the above-mentioned limitations, our data show that RATP in selected patients is non-inferior to OTP regarding occurrence of severe post-operative complications. Therefore, this study contributes to define the role of robotic assistance in very complex procedures, such as total pancreatectomy.

We wish to underscore that the 25 RATPs reported herein constitute approximately 6% of our experience with robotic pancreatic resections and < 2% of our overall volume of pancreatic resections during the study period. Reproducibility of our results in centers with lower volumes of activity remains to be established.

## Supplementary Information

Below is the link to the electronic supplementary material.

Supplementary file1 (MP4 326828 KB)

## Data Availability

All materials are available upon request.
